# Targeting the pMHC–TCR Interaction: Molecular Strategies and Therapeutic Potential in Autoimmunity

**DOI:** 10.3390/ijms27083622

**Published:** 2026-04-18

**Authors:** Alina M. Nechaeva, Azad E. Mamedov, Leyla A. Ovchinnikova, Mariya Y. Zakharova

**Affiliations:** Shemyakin-Ovchinnikov Institute of Bioorganic Chemistry RAS, 117997 Moscow, Russia

**Keywords:** antigen-specific immunotherapy, peptide–MHC complexes, autoimmune diseases, self-tolerance, immune tolerance induction, T cell receptor

## Abstract

Autoimmune diseases arise from the failure of self-tolerance. The recognition of self-antigen peptide–MHC (pMHC) complexes by the T-cell receptor (TCR) is the fundamental event triggering autoimmune pathogenesis. While traditional immunosuppressants provide broad systemic effects, they often compromise global immunity. Emerging molecular strategies aim to selectively disrupt the trimolecular complex—comprising the TCR, the antigenic peptide, and the MHC molecule—to induce antigen-specific tolerance. This review highlights the pMHC–TCR interaction as the primary molecular checkpoint for antigen-specific intervention. We discuss the structural basis of these interactions and their potential to redefine the therapeutic landscape for autoimmune diseases (ADs). We examine the molecular drivers of tolerance breakdown—including genetic susceptibility, molecular mimicry, post-translational modifications (PTMs), and ectopic MHC II expression—that shape the autoreactive T-cell landscape. This review examines current advancements in biological and pharmacological interventions, such as pMHC-decorated nanoparticles and soluble pMHC, to reprogram pathogenic T-cell response. We also explored CAR-T therapy strategies for autoimmune diseases, such as CAR-Treg, designed to precisely modulate pMHC-TCR signaling. Collectively, these precision interventions in immunological synapse assembly during autoimmune response are considered the basis for safer, antigen-specific immunotherapy capable of restoring self-tolerance without global immunosuppression.

## 1. Introduction

ADs are characterized by a profound loss of self-tolerance, leading to the activation of autoreactive T and B lymphocytes and the subsequent production of autoantibodies that target host tissues [1]. This loss of self-tolerance results in a persistent immune response against endogenous antigens, driving chronic systemic inflammation. Although many autoimmune diseases are considered rare (the number of patients is about 10% of the world’s population [2]), their collective incidence rose substantially between 1985 and 2015, reflecting a broader trend of increasing immune-mediated pathology (by 19.1% per year) [3]. Furthermore, an additional post-COVID-19 surge in autoimmunity was documented between 2019 and 2021, with reports indicating a further 1–2% increase in global incidence [4]. Autoimmune disorders are characterized by extensive comorbidities, with cardiovascular, respiratory, and endocrine diseases contributing to significantly higher mortality rates in these patient populations [5]. It is assumed that this pattern may result not only from systemic inflammation, but also from the use of immunosuppressive therapy.

Currently, systemic immunosuppression remains the mainstay of treatment for autoimmune diseases. The first immunosuppressive drugs (in the 1940s–1950s) were glucocorticosteroids and cytotoxic agents such as cyclophosphamide [6]. Cyclophosphamide exerts its immunosuppressive effect by inhibiting DNA replication, primarily through the formation of crosslinks between N7 atoms of guanine residues [7]. Failure to complete DNA replication due to stable crosslinking prevents S-phase progression, leading to the apoptotic depletion of rapidly dividing immune cells [8,9]. The non-selective cytotoxic profile of cyclophosphamide leads to the inhibition of all rapidly proliferating cells, targeting not only autoreactive lymphocytes but also hematological progenitors in the bone marrow. This mechanism accounts for its significant hematologic toxicity, characterized by a high incidence of neutropenia (73%) and leukopenia (70%) [10]. Similarly, systemic corticosteroids—another class of broad-spectrum immunosuppressants—exert their effects as ligands for the glucocorticoid receptor (GR). Upon activation, the ligand–receptor complex translocates into the nucleus, where it functions as a transcription factor to upregulate the expression of various anti-inflammatory proteins (IL-10, lipocortin-1, and IκBα). Simultaneously, the suppression of NF-κB, STAT, and AP-1 signaling results in a global downregulation of pro-inflammatory cytokines and chemokines, which is central to the therapeutic efficacy of corticosteroids [11,12,13]. However, the binding of glucocorticosteroids to the GR also upregulates the expression of key gluconeogenic enzymes in hepatocytes. This metabolic shift accounts for the high clinical incidence of secondary hyperglycemia (32%) and the development of steroid-induced diabetes mellitus (19%) [14]. Despite the proven efficacy of these drugs, the lack of selectivity for immune cells in general and for autoreactive cells in particular leads to a high risk of severe adverse effects.

The subsequent evolution of autoimmune therapy involved the design of more selective immunosuppressive strategies, specifically focusing on agents that target key components of the adaptive immune system, namely T and B cells. For example, cyclosporine A acts by inhibiting calcineurin, which in turn blocks activation of the transcription factor NFAT and reduces the expression of cytokines required for T-cell activation and proliferation [15]. The increased specificity of this drug stems from the restricted cellular distribution of calcineurin, in contrast to the ubiquitous expression of the GR. Despite its relative selectivity for immune cells, calcineurin is also functionally active in the nervous system [16], which explains the neurotoxic effect of cyclosporine observed in 10–28% of cases [17]. More refined immune-specific therapies are represented by monoclonal antibodies, which selectively target surface receptors or neutralized key pro-inflammatory cytokines. Drugs such as anti-TNF (adalimumab, infliximab, golimumab) and anti-IL17/23 (secukinumab/ustekinumab) bind signaling molecules and thereby block proinflammatory signaling pathways, suppressing inflammation on protein level but not on gene expression level [18]. A prominent example of an agent targeting specific cell surface antigens is rituximab, a chimeric monoclonal antibody directed against CD20. Because CD20 expression is limited to pre-B and mature B cells, the drug’s cytotoxic effect is highly specific, leaving other immune cells unaffected. Upon binding to the CD20 antigen, the antibody specifically induces cellular complement-dependent cytotoxicity [19,20]. Common side effects of rituximab therapy include infusion reactions (25%) caused by cytokine release from lysing B cells [21] and hypogammaglobulinemia (9.5%) [22]. Targeted depletion of specific TCR families, such as the TRBV9^+^ clones associated with ankylosing spondylitis, is now possible with the humanized antibody Seniprutug. This approach allows for the elimination of disease-driving T cells with minimal impact on overall immune surveillance [23].

The most promising frontier for specific immunosuppression is the targeting of the TCR–autoantigen–MHC complex. Intercepting this initial recognition event provides a pathway to antigen-specific tolerance, representing the next logical step beyond broad B- or T-cell depletion. Precision therapies centered on pMHC represent one of the most promising trends in autoimmunity. By shifting the focus from global suppression to antigen-specific modulation, these agents aim to re-establish tolerance with minimal off-target effects. Selective topological blockade of pMHC complexes, elimination of autoreactive T cells, or switching their activity toward a regulatory phenotype using therapeutic pMHC- based agents may improve the quality and duration of life of patients with autoimmune diseases and also reduce the number and severity of adverse effects [24]. A significant gap remains between the rational design of antigen-specific therapies and their successful translation into late-stage clinical development [25]. Here, we delineate the molecular pathways driving auto reactivity from pMHC assembly to TCR engagement, followed by a comprehensive overview of precise molecular approaches and therapies aimed at restoring self-tolerance.

## 2. The pMHC–TCR Interaction and Its Role in Autoimmune Pathogenesis

### 2.1. Antigen Presentation by MHC

The molecular recognition of self-peptides in complex with MHC molecules by T lymphocytes is a pivotal event in the etiology of autoimmune diseases. This process involves the proteolytic processing of endogenous and exogenous proteins, followed by the loading of these fragments onto MHC molecules within endosomal compartments or the endoplasmic reticulum for subsequent display on the cell surface [26]. T-cell activation is initiated by the molecular recognition of the pMHC complex by the TCR, a process stabilized by the recruitment of CD4 or CD8 coreceptors to the invariant domains of MHC class II or I, respectively. To elicit a robust immune response, this primary signal must be integrated with costimulatory signaling, primarily mediated by the CD28-CD80/86 interaction [27]. The combination of these molecular contacts forms a functional immunological synapse that provides precise control over T-cell differentiation [28].

MHC class I molecules facilitate the presentation of endogenous peptides derived from cytosolic proteins and intracellular pathogens to CD8^+^ T cells. These antigens are primarily processed by the proteasome before being translocated into the endoplasmic reticulum [29]. In contrast, MHC class II molecules specialize in the presentation of exogenous proteins acquired via endocytosis, which are proteolytically cleaved within lysosomal compartments for display to CD4^+^ T cells [30]. While MHC class I is constitutively present on most nucleated cell types, MHC class II is a hallmark of professional antigen-presenting cells (APCs)—macrophages, B cells, and dendritic cells. However, the inflammatory milieu can trigger the de novo expression of MHC class II on non-professional APCs (such as epithelial cells, endothelial cells, fibroblasts, and others), thereby expanding the repertoire of cells capable of activating CD4^+^ T-cell responses during chronic autoimmune inflammation [31,32].

Most autoimmune predispositions are linked to specific HLA-DR/DQ alleles, making the MHC-II groove a primary target for blockade.

### 2.2. Molecular Mechanisms of T-Cell Activation

Under physiological conditions, the introduction of foreign antigens via MHC presentation triggers the adaptive immune cascade. Specifically, for CD8^+^ T cells, this stimulation initiates a robust cytotoxic response characterized by clonal expansion. During this process, activated cells undergo divergent differentiation into either functional cytotoxic T lymphocytes (CTLs), responsible for immediate effector activity, or long-lived memory T cells for persistent immunosurveillance [33]. Short- lived CTLs identify target cells via specific MHC I–antigen recognition. Upon the assembly of the immunological synapse (trimolecular complex), they execute target cell apoptosis [34]. CD8^+^ memory T cells persist in the organism even after elimination of infected cells and provide a rapid immune response upon re-encounter with the same antigen [35]. Upon activation, naïve CD4^+^ T cells undergo lineage-specific differentiation into effector (Th and Tfh cells) and regulatory populations (Tregs). The latter group is further distinguished by the presence of FoxP3^+^ Tregs and the FoxP3^−^ Tr1 subset (IL-10^+^ type 1 regulatory T cells) [36]. Effector CD4^+^ T cells orchestrate immune responses by secreting distinct cytokine profiles [37,38].

Tregs ensure immunological tolerance by modulating autoreactive or excessive responses. One primary mechanism involves the high-affinity inhibitory receptor CTLA-4, which competitively outbinds CD28 for CD80/CD86 ligands on APCs, thereby depriving effector cells of essential co-stimulation. Additionally, Tregs facilitate competitive IL-2 sequestration; by expressing high-affinity CD25 (IL-2Rα), they effectively deplete the local cytokine pool, starving effector populations and limiting their proliferation and survival [39,40]. Beyond metabolic disruption, Tregs exert direct immunosuppression on Th cells, CD8^+^ T cells, B cells, and DCs via the secretion of IL-10 and TGF-β [41,42]. Given their potent capacity to modulate the immune landscape, Treg-based therapies are currently being explored for both autoimmune disorders and oncological intervention.

The outcome of TCR–pMHC interactions is highly context-dependent; upon encountering self-antigens during maturation, T cells follow a specialized differentiation pathway known as central tolerance. While foreign antigens trigger an effector cascade, the presentation of self-antigens is governed by a fundamentally different developmental paradigm aimed at maintaining tolerance. Within the thymic microenvironment, the initial encounter of a developing T cell with self-pMHC complex facilitates the establishment of central tolerance [43]. This process is dictated by the binding affinity of the TCR for self-antigens. It begins with positive selection, which preserves only those T cells capable of functional interaction with host MHC molecules. This is followed by negative selection, wherein clones exhibiting dangerously high affinity for self-pMHC complexes are eliminated via apoptosis. This stringent thymic filtering serves to purge potentially pathogenic autoreactive clones before they egress into the systemic circulation [44]. Nevertheless, this process is incomplete; evidence suggests that up to 30% of self-reactive T cells bypass thymic deletion and reach the periphery [45]. Consequently, the robust establishment of peripheral tolerance becomes essential for maintaining immunological silence toward host tissues [46].

### 2.3. Peripheral Tolerance Induction

Peripheral tolerance serves as a secondary check to neutralize mature autoreactive lymphocytes that have bypassed central selection in the thymus or bone marrow. This systemic regulation is achieved through several distinct mechanisms: anergy, a state of functional hypo responsiveness resulting from TCR engagement in the absence of co-stimulatory signals; clonal deletion, the induction of apoptotic pathways in self-reactive cells; and active suppression, primarily orchestrated by Tregs via inhibitory receptors and anti-inflammatory cytokine secretion. In addition, immunological ignorance acts as a passive barrier, keeping autoreactive clones quiescent due to sub-threshold antigen concentrations or their sequestration within immune-privileged sites [46].

**Figure 1:**
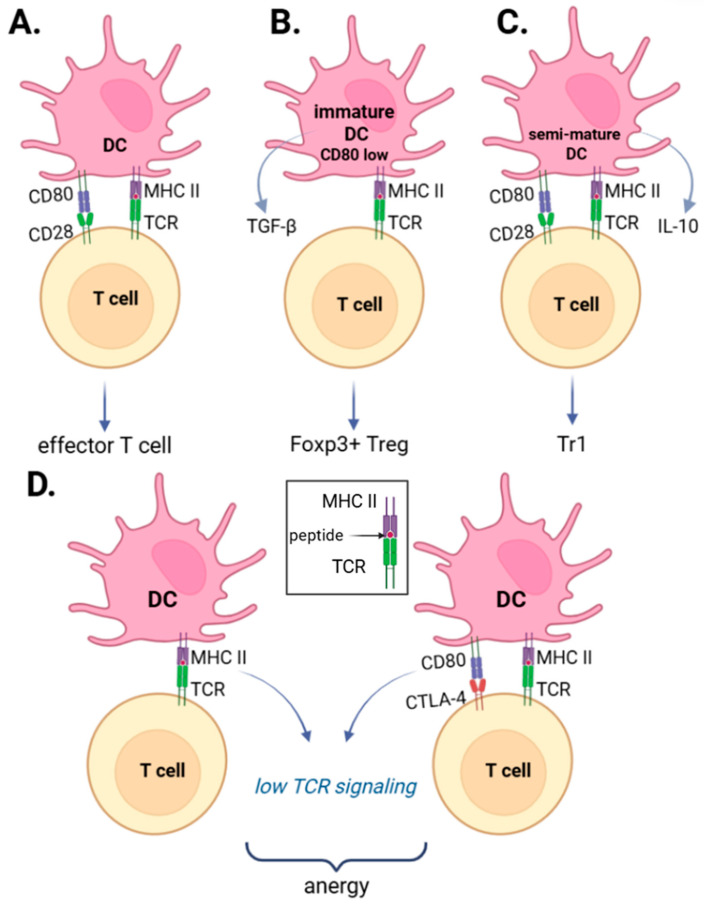
Molecular Mechanisms of Peripheral CD4^+^ T-Cell Tolerance and Phenotypic Differentiation. Schematic representation of T-cell fate determination based on the pMHC–TCR signaling context. (**A**) Canonical activation: Robust CD28–CD80/86 co-stimulation (antigen presentation by mature DCs) promotes the differentiation of naive T cells into pro-inflammatory effector populations. (**B**) Treg induction: Low level of CD80–CD28 co stimulation (antigen presentation by tolerogenic or immature DCs) in the presence of TGF β results in differentiation into FoxP3^+^ Treg. (**C**) Tr1 differentiation: Moderate co-stimulatory signaling (antigen presentation by semi-mature DCs) in the presence of IL-10 promotes the development of Type 1 regulatory (Tr1) cells. (**D**) Functional anergy: The absence of co-stimulation or the competitive engagement of the CTLA-4–CD80/86 inhibitory axis induces a state of functional anergy.

CD28-mediated co-stimulation serves as a critical checkpoint for peripheral tolerance (Figure 1). In the absence of robust CD80/CD86 signaling, TCR engagement drives T cells into anergy, characterized by impaired IL-2 production and arrested proliferation [47]. This inhibitory state is further reinforced by CTLA-4, which outcompetes CD28 for ligand binding, thereby actively antagonizing activating signals [48]. Furthermore, the co-stimulatory context dictates the functional polarization of naïve CD4^+^ T cells into regulatory subsets. Foxp3^+^ Tregs typically differentiate upon interaction with immature dendritic cells (imDCs), exhibiting low CD80/CD86 density, providing the sub-threshold co-stimulation necessary for induction. This transition is highly dependent on the cytokine microenvironment, where high concentrations of TGF-β and IL-2 are required to stabilize Foxp3 expression. In contrast, Tr1 cell differentiation is favored during interactions with semi-mature DCs (smDCs) or imDCs under moderate co-stimulatory conditions, traditionally associated with IL-10 signaling [49]. However, alternative models suggest that Tr1 induction may rely more heavily on chronic, repetitive TCR stimulation under limited co-stimulation rather than a strict IL-10 requirement [50]. Finally, the total absence of CD28 signaling and IL-2 leads to the passive death of naïve cells, while chronic re-activation—often via the Fas–FasL axis—triggers the deletion of effector populations [51]. This complex mechanism of peripheral tolerance formation (Figure 1) is used to develop therapeutic agents, which are described in detail in the following sections.

As the important master checkpoint of adaptive immunity, the pMHC–TCR interaction serves as the primary filter between quiescence and activation, where a loss of recognition fidelity triggers the breakdown of tolerance. This fundamental signal is integrated into a regulatory network where the canonical CD28–CTLA-4 axis establishes the response amplitude by either amplifying proximal signaling or competitively aborting autoreactive priming. Further refinement is provided by secondary modulators, for example, PD-1 [52] and Siglec-7/-9 [53], which recalibrate TCR sensitivity via SHP-1/2 recruitment [54,55] or ITIM signaling [56]. Ultimately, while the pMHC–TCR axis remains the dominant control node, the hierarchy of subsequent checkpoints dictates the progression toward a systemic autoimmune phenotype.

### 2.4. Genomic Drivers of Self-Tolerance Defects

A breakdown in immune homeostasis can be triggered by a genetic predisposition that disrupts mechanisms of self-tolerance [57]. Immune tolerance to self-antigens may be genetically impaired at the stage of negative selection of T lymphocytes in the thymus. For example, in the case of mutations in the *AIRE* (Autoimmune Regulator) gene, which controls the expression of tissue-specific antigens by thymic epithelial cells [57]. Defects in the apoptotic machinery, caused by mutations in the Fas and FasL genes, can also allow autoreactive T-cell clones to survive [58]. Peripheral tolerance may be compromised by reduced function and numbers of regulatory T cells, which can be associated with mutations in the Foxp3 gene, as well as by dysregulated immune control of effector T cells due to mutations in IL-2 and CTLA-4 [59].

Extensive evidence indicates that genetic susceptibility to numerous autoimmune pathologies is primarily linked to the inheritance of specific MHC alleles. The binding affinity of a given MHC variant for particular antigenic motifs is a critical determinant of whether a self-peptide will be presented for TCR recognition [60]. A classic example is rheumatoid arthritis (RA), where approximately 90% of patients harbor the HLA-DRB1*0401, *0404, or 0101 alleles* [61]. This strong clinical association is attributed to a conserved amino acid sequence—the ‘shared epitope’—located at positions 70–74 of the DRβ1 chain. Positioned within the P4 pocket of the antigen-binding groove, this motif facilitates the preferential presentation of arthritogenic peptides, thereby driving the expansion of an autoreactive T-cell repertoire [62].

### 2.5. Mechanisms of Peripheral Tolerance Breakdown

Exogenous environmental triggers can significantly contribute to the breakdown of self–non-self-discrimination. When a pathogenic protein exhibits structural homology with host proteins, it can lead to cross-reactivity. In this scenario, a single MHC variant can present both foreign and self-peptides, which are subsequently recognized by the same TCR—a phenomenon termed molecular mimicry [63,64].

Following an infectious challenge, the pro-inflammatory cytokine milieu serves as a critical pathogenic driver for the induction of autoreactive responses. A hallmark of this process is the aberrant upregulation of MHC II expression within inflammatory foci. Elevated local concentrations of interferon-gamma (IFN-γ) trigger the activation of the CIITA promoter—the master transcriptional regulator of the MHC class II gene cluster—in non-professional antigen-presenting cells [65]. Consequently, host cells that are typically immunologically ‘invisible’ to CD4^+^ T helper cells begin to display self-peptides, effectively functioning as ectopic APCs [66]. In Hashimoto’s thyroiditis, for example, thyroid epithelial cells undergo this phenotypic shift under inflammatory stress, transitioning from passive targets to active stimulators of T-lymphocyte infiltration [67].

A critical consequence of localized inflammation is bystander activation, wherein the activation threshold for T cells is significantly lowered. The abundance of pro-inflammatory cytokines (e.g., IL-1, IL-6, and TNF-α) and damage-associated molecular patterns (DAMPs), such as heat-shock proteins (HSPs) or cell-free DNA, drives the robust upregulation of CD80/86 on local APCs. In this hyper-stimulatory milieu, autoreactive T cells possessing only marginal affinity for self-pMHC complexes receive sufficient co-stimulatory signals to bypass normal checkpoints, leading to their clonal expansion and functional activation [68].

Moreover, many autoantigens that are normally sequestered inside cells or in immune-privileged organs with blood–tissue barriers (eyes, brain, testes) and that T cells have never encountered during their “education” become accessible to APCs during inflammation, which causes extensive cell death (necrosis, NETosis) and barrier disruption. This release leads to the systemic accumulation of intracellular material, which can be captured and processed by APCs for MHC II presentation [69,70]. This massive display of self-antigens within a pro-inflammatory milieu frequently triggers epitope spreading. This phenomenon involves a functional shift in the T-cell response, transitioning from a dominant pathogen-specific focus to the recognition of self-epitopes presented by both professional and non-professional APCs. This process facilitates the emergence of neo-epitopes within a single autoantigenic protein, fueling the diversification of the autoreactive T-cell repertoire and the chronic propagation of the immune response [71].

Thus, presentation of self-peptides on MHC II in inflamed tissues converts a local protective process into a self-perpetuating cycle of tissue destruction, preventing spontaneous resolution of inflammation and ultimately leading to the development of full-blown autoimmune disease [72].

The breakdown of self-tolerance is intrinsically linked to antigenic variability. Factors such as PTMs and the formation of protein complexes create neoantigens that the immune system fails to recognize as ‘self,’ thereby serving as primary initiators of autoimmune pathology. Pathogenesis in antiphospholipid syndrome (APS) involves neoepitopes generated through β2GPI interaction with platelet factor 4 (PF4). The resulting immunoreactive complexes trigger platelet-driven thrombosis, highlighting a complex antigenic landscape that exceeds the targeting of native β2GPI alone [73].

A striking example of PTM is citrullination of proteins in rheumatoid arthritis, associated with hyperactivation of peptidyl arginine deiminase (PAD). Conversion of arginine to citrulline has been shown to increase peptide affinity for MHC molecules carrying the shared epitope, which promotes their presentation to CD4^+^ T cells [74]. Other types of PTMs also occur, such as carbamylation and deamidation. These processes generate “neoepitopes” that were not previously presented to T cells in the thymus during selection. A relevant example is APS, where carbamylation of β2GPI may create additional antigenic determinants beyond native β2GPI [75,76]. Extracellular vesicles may further modulate β2GPI-driven pathogenic pathways in APS by exposing antigenic targets and reinforcing endothelial prothrombotic activation [77].

An autoimmune response can also arise when previously sequestered antigens come “into view” of the immune system. In systemic lupus erythematosus, the immune response targets fragments of double-stranded DNA and chromatin that remain in the extracellular space due to defects in the clearance of apoptotic material [78]. As a result, T cells recognize such modified self-molecules as foreign. At the site of inflammation (for example, a joint in arthritis), MHC class II molecules present precisely these altered peptides.

**Figure 2:**
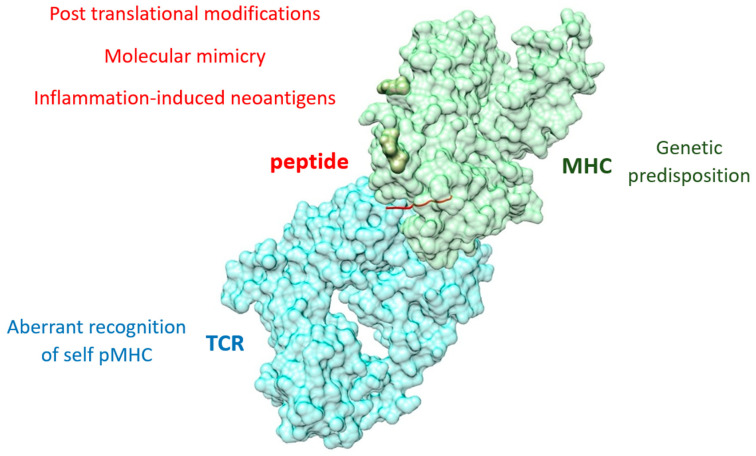
Molecular determinants of pMHC–TCR synapse dysfunction in autoimmunity: Genetic predisposition through specific MHC (green) polymorphism of risk alleles influences the presentation of autoantigenic peptides (red). The conversion of self-proteins into pathogenic neoantigens via PTMs and molecular mimicry provides high-affinity ligands for autoreactive TCRs (blue). The resulting breakdown in the pMHC–TCR trimolecular complex specificity bypasses peripheral tolerance and initiates tissue destruction. The model of the pMHC–TCR Complex is represented by a Tfh TCR and an aggrecan peptide presented by MHC II. Rendered from PDB entry 7RDV [80].

The fundamental molecular insights into peripheral tolerance breakdown are now being leveraged to engineer antigen-specific therapeutics designed for its restoration. By translating the principles of immune checkpoints and synapse dynamics into programmable platforms, such as pMHC-nanoparticles and Tolerogenic Vaccines, researchers aim to re-induce anergy and active suppression within the autoreactive repertoire. Thus, targeting the individual components of the pMHC II–TCR complex offers a promising avenue for the development of tolerance-inducing biologics. These strategies aim to selectively suppress pathogenic responses while sparing protective immunity, providing a pathway to halt the progression of autoimmune diseases without the harmful effects of broad-spectrum immunosuppressants [79] (Figure 2).

## 3. pMHC-Based Strategies for Restoring Peripheral Tolerance

Various therapeutic modalities targeting the pMHC–TCR synapse are currently at distinct stages of preclinical and clinical development, with the primary objective of restoring immunological tolerance to self-antigens. As previously discussed, the TCR recognition of the pMHC complex is a decisive signaling event; the subsequent fate of T cells—from proinflammatory activation to functional anergy or differentiation into regulatory T cells (Treg)—is primarily determined by additional costimulatory signals and the local cytokine environment. The strategies detailed in this section modulate the co-stimulatory context to shift the immune response toward antigen-specific suppression of the autoreactive cascade and the induction of immunological tolerance.

Two fundamentally distinct therapeutic strategies have emerged in this field. The first utilizes APC-independent molecular platforms, employing soluble pMHC complexes, free antigens, or advanced delivery systems such as nanoparticles, liposomes, and other biocompatible carriers. The second approach relies on the presentation of autoantigens by tolerogenic dendritic cells (tolDC), which are characterized by a low expression of CD80/CD86 co-stimulatory molecules [81].

### 3.1. Soluble Antigen Immunotherapy

The therapeutic efficacy of soluble antigen-mediated immunotherapy is predicated on high-dose administration, which triggers functional T-cell anergy or drives a regulatory immune response [82]. The use of soluble autoantigens has evolved from the administration of whole proteins (insulin, myelin basic protein (MBP)) to the design of short synthetic peptides (12–20 amino acids). Current strategies focus on autoantigenic peptides which, due to their solubility and bioavailability, can efficiently compete for binding within the MHC II peptide-binding groove on the surface of immature antigen-presenting cells, thereby suppressing the autoimmune cascade in individuals carrying specific risk alleles [83] (Table 1). Several peptide-based candidates are undergoing preclinical and clinical evaluation, including fragments of insulin for Type 1 Diabetes [84,85] (Nasal Insulin-Phase III clinical trial, NCT00223613) and MBP-derived fragments for Multiple Sclerosis [86,87] (ATX-MS-1467-Phase II clinical trial, NCT01973491). Numerous studies highlight the HLA-restricted clinical response of these agents, as therapeutic benefits are predominantly observed in patients carrying specific high-risk HLA-II alleles (MBP8298-Phase III clinical trial, NCT00468611) [88,89]. This association confirms that these peptides modulate the autoimmune cascade specifically by targeting the antigen presentation pathway.

Extensive research has focused on altered peptide ligands (APLs) to induce immunological tolerance. Being analogs of native antigenic determinants, APLs contain single amino acid substitutions in the region of contact with TCR, which allows modulation of binding affinity and subsequent CD3 phosphorylation pattern [90]. This molecular reprogramming can induce T-cell anergy or drive its phenotype shift from a pro-inflammatory Th1/Th17 profile toward a regulatory Th2 response [91]. Beyond linear peptides, cyclic APLs have emerged as promising candidates for the treatment of multiple sclerosis (cyclo(87–99)(Ala91,Ala96)MBP87–99-preclinical study) [92], (Cyclic MOG_35-55_-preclinical study) [93].

The most prominent clinical implementation of this strategy is glatiramer acetate (Copaxone—approved NCT00097188), which functions as a universal APL for myelin-reactive T cells in multiple sclerosis, promoting a protective Th2 shift and suppressing neuroinflammation [94]. Another significant development involves insulin B:9–23-based APLs (e.g., NBI-6024-Phase I clinical trial, NCT00873561) for type 1 diabetes, engineered to restore immunological tolerance to pancreatic β-cells [95]. Furthermore, Jusvinza (Phase III clinical trial, RPCEC00000404), a modified peptide derived from heat shock protein 60 (HSP60), has been shown to downregulate pro-inflammatory cytokines and bias the immune response toward a regulatory phenotype in rheumatoid arthritis [96]. Despite their therapeutic promise, the clinical application of APLs necessitates molecular precision to minimize the risks of cross-reactivity or potential disease exacerbation. Thus, the clinical evaluation of APLs in multiple sclerosis unexpectedly triggered severe disease flares; as detailed in an erratum published by the authors in Nature Medicine, this outcome was driven by the cross-activation of encephalitogenic T-cell responses rather than the intended induction of immune tolerance [97].

Peptide-based immunotherapy provides a precise means of inducing antigen-specific tolerance without global immunosuppression [98], yet several hurdles remain. While these strategies effectively promote regulatory T cells or clonal anergy, their clinical translation is hindered by the metabolic instability of peptide-based drugs and their strict HLA restriction. Because therapeutic efficacy is fundamentally contingent upon the patient’s individual HLA haplotype and the presence of specific risk alleles, this genetic diversity results in significant response variability, thereby constraining the broad clinical utility of peptide therapies [26]. Furthermore, a significant translational gap persists; while APLs show promise in experimental models of systemic autoimmunity, human clinical data remain fragmentary and frequently fail to replicate the robust responses observed in preclinical settings [99].

### 3.2. Soluble pMHC Complex-Based Immunotherapy

The production of soluble pMHC complexes is slightly more demanding than that of soluble antigens, primarily due to the additional biotechnological steps required to generate stable MHC variants. These constructs necessitate either exogenous peptide loading or covalent linkage to the MHC scaffold. To streamline this process, specialized single-chain chimeric molecules, termed recombinant TCR ligands (RTLs), have been engineered; these consist of a single polypeptide chain comprising the antigenic peptide fused to the β chain and a chain domain of the MHC class II molecule [100,101,102]. Furthermore, several studies have demonstrated that pMHC dimers and higher-order multimeric complexes exhibit enhanced therapeutic efficacy compared to their monomeric counterparts [103,104].

The mechanism of action of soluble pMHC complexes is based on the direct and selective delivery of signals to specific T-cell populations, bypassing the requirement for APC-mediated activation and inflammatory co-stimulation. Since these complexes are administered in a soluble monomeric or multimeric form rather than presented on APC cell membrane, the essential CD28–CD80/CD86 co-stimulatory axis is not engaged. This lack of secondary signaling induces functional T-cell anergy or drives differentiation toward a regulatory T-cell (Treg) phenotype. Early proof-of-concept studies at the end of the 20th century demonstrated that soluble pMHC molecules carrying MBP peptide fragments could suppress the development of experimental autoimmune encephalomyelitis (EAE) in murine models [105], while complexes utilizing acetylcholine receptor fragments were effective in experimental myasthenia gravis [106]. In a clinical setting, however, a phase I trial of AG284 (a soluble HLA-DR2:MBP84–102 complex) failed to show significant differences compared to placebo [107]. This lack of efficacy may be attributed to the low intrinsic avidity of the pMHC–TCR interaction or a sub-therapeutic local concentration of the complexes, leading to insufficient T-cell engagement. More recent therapeutic approaches utilize MHC tetramers to enhance binding stability (Table 2).

Compared to free peptides, pMHC-multimers provide superior therapeutic precision by leveraging high-avidity interactions to engage low-affinity and low frequency autoreactive T cells [111]. While free peptides require uptake and processing by APCs, pMHC-multimers signal T cells directly, enabling the active reprogramming of effectors into Tr1 cells [24]. However, pMHC-multimers face steeper translational hurdles due to extreme HLA-restriction, high production costs, and the technical complexity of maintaining stable recombinant MHC complexes [24,112].

### 3.3. Antigen-Conjugated Nanoparticle Immunotherapy

**Figure 3:**
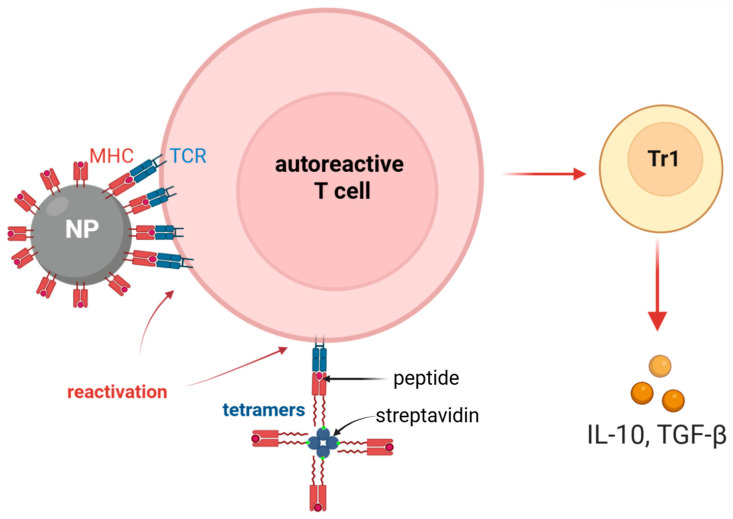
Schematic representation of the antigen-specific reprogramming of autoreactive T cells into Tr1 cells: In the absence of co-stimulatory signals, the engagement of the TCR by pMHC–nanoparticles or pMHC tetramers bypasses canonical effector activation. This suboptimal signaling facilitates a phenotypic shift toward a Treg-mediated suppressive response, effectively inhibiting the autoimmune cascade.

Currently, the development of therapeutic agents has shifted toward pMHC complexes conjugated to liposomes or nanoparticles, primarily due to their enhanced biochemical stability. Most nanoparticle- and liposome-based platforms function by inducing the differentiation of effector T cells into IL-10-secreting regulatory T cells. These formulations may consist of nanoparticles coated with multimeric pMHC complexes or, alternatively, with isolated antigen molecules in the absence of the MHC scaffold. When previously activated T cells re-encounter antigens displayed on these nanoparticles in the absence of co-stimulatory signals—a mechanism analogous to MHC tetramer-mediated signaling—they bypass full activation, resulting in functional anergy or regulatory Tr1 differentiation (Figure 3) [113].

The surface density of pMHC complexes (valence) represents a critical parameter in the molecular design of these therapeutics. Nanoparticles characterized by insufficient pMHC valence typically exhibit sub-optimal biological activity [114]. Conversely, an excessively high pMHC density can trigger aberrant T-cell activation, potentially driving a robust pro-inflammatory effector response [115,116]. Such unintended activation must be strictly avoided, as it counteracts the therapeutic goal of inducing antigen-specific tolerance. Thus, multivalent pMHC display on nanoparticles provides sustained TCR stimulation in the absence of CD28 co-stimulation, biasing the fate of previously activated autoreactive CD4^+^ T cells toward IL-10^+^ Tr1 differentiation or the establishment rather than effector reactivation [24].

Peptide–MHC-based nanoparticle medicines represent a sophisticated therapeutic frontier, offering high biochemical stability and precise control over antigen valence to induce antigen-specific regulatory T cells without compromising global immunity. However, their clinical translation is hindered by significant technological and biological challenges: these include the rigorous requirement for accurately defined, clinically relevant autoantigens and the necessity for highly selective delivery to target cells while avoiding off-target accumulation or unintended immune activation. Furthermore, substantial obstacles regarding industrial-scale production, high manufacturing costs, and long-term formulation stability remain to be addressed to ensure their broader clinical viability [117,118].

### 3.4. Cell-Based Immunotherapy via Tolerogenic Autoantigen Presentation

Immunotherapeutic strategies that exploit the distinct tolDC phenotype in conjunction with antigen-specific presentation constitute a dedicated field of study (Table 3). Tolerogenic DCs are characterized by low expression of the co-stimulatory molecules CD80/CD86 and by a distinctive cytokine profile: IL-10^+^ TGF-β^+^ IL-27^+^. This specialized signaling milieu recalibrates the T-cell response at the antigen-presentation interface, driving either functional anergy or the induction of a regulatory phenotype [119].

**Figure 4:**
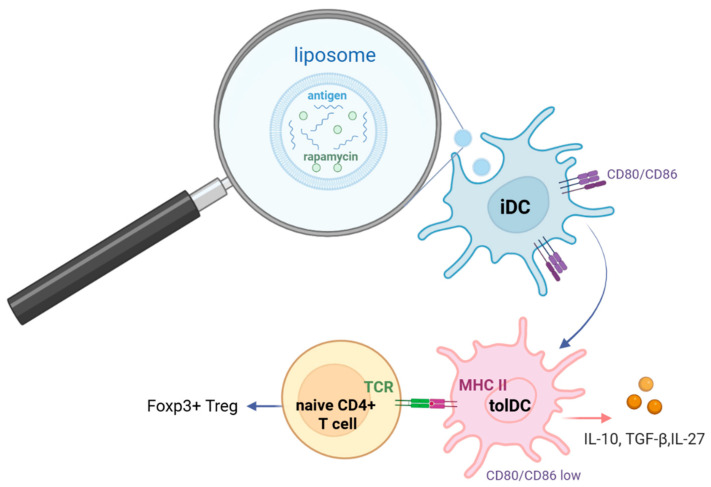
TolDC-Mediated Induction of Regulatory T Cells. Schematic representation of the tolerogenic reprogramming of iDCs. Liposomes containing both a specific autoantigen and a tolerant substance (e.g., rapamycin) are engulfed by phagocytosis. Intracellular rapamycin delivery modulates the DC phenotype, promoting a tolerogenic state characterized by downregulated CD80/CD86 expression and the secretion of IL-10, TGF-β, and IL-27. Subsequent presentation of the autoantigen to naive T cells within this specialized signaling milieu drives their differentiation into Tregs and Tr1 cells.

TolDC populations can emerge endogenously in humans (e.g., DC-10, smDC) or be generated ex vivo by treating immature iDCs with pharmacological agents such as vitamin D3, rapamycin, or the cytokines IL-10 and TGF-β [120]. Specifically, rapamycin inhibits the mTOR signaling pathway, which subsequently suppresses IL-12 expression while concurrently upregulating IL-10 and TGF-β production [121]. Current tolerogenic modalities often utilize liposomal platforms that co-encapsulate both the target antigen and a tolerizing agent (Figure 4). Alternative strategies involve the adoptive transfer of ex vivo engineered tolDCs loaded with the relevant autoantigen (Table 2). Furthermore, antigen-specific tolerance can be induced via nucleic acid constructs (DNA/RNA) designed to drive the coordinated expression of the autoantigen alongside IL-10 and TGF-β within the dendritic cell. In all the approaches described above, the disease-relevant autoantigen is presented by tolDCs via MHC class II under conditions of minimal CD80/CD86 co-stimulation and an anti-inflammatory cytokine milieu (IL-10/TGF-β/IL-27). This configuration delivers a sub-immunogenic TCR signal to autoreactive CD4^+^ T cells. Chronic exposure to this tolerogenic pMHC environment triggers anergy or clonal deletion while simultaneously driving the differentiation of FoxP3^+^ and Tr1 regulatory T cells, thereby actively restoring antigen-specific peripheral tolerance [122,123,124].

Cell-based strategies utilizing tolerogenic dendritic cells (tolDCs) offer significant therapeutic potential by enabling antigen-specific modulation and favoring the induction of regulatory T cells over broad immunosuppression. Early-phase clinical trials across multiple autoimmune pathologies—including multiple sclerosis, type 1 diabetes, and rheumatoid arthritis—have already confirmed their favorable safety profile and initial biological activity. Nevertheless, their clinical translation remains hampered by the inherent complexities of patient-specific manufacturing and the high degree of inter-donor variability regarding tolDC functional stability in the context of established autoimmunity. Furthermore, the lack of standardized protocols for antigen loading, optimal dosing, and administration routes remains a critical barrier, significantly escalating the cost and logistical complexity of these personalized interventions [125].

**Table 3:** Immunotherapeutic Agents based on autoantigen presentation by tolDC.

Drug Name	Disease	Antigen	Origin of the Antigen	Composition	Mechanism of Action	Development Stage
Liposomes encapsulating BDC2.5 mim + calcitriol [126]	Type 1 diabetes	BDC2.5 mimotope	Mimotope peptide that mimics the structure of a chromogranin A fragment (BDC2.5mim)	Phosphatidylcholine/phosphatidylglycerol liposomes loaded with antigen and calcitriol	Induction of differentiation of CD4^+^ T cells into Foxp3^+^ Treg and Tr1	Preclinical studies, mouse model
SEL-212 (Pegadricase + ImmTOR) [127]	Chronic refractory gout	SEL-037	PEGylated uricase (pegadricase)	Combination of PEGylated uricase with ImmTOR nanoparticles (administered separately). ImmTOR are PLA/PLA-PEG nanoparticles encapsulating rapamycin [128]	Induction of differentiation of CD4^+^ T cells into Foxp3^+^ Treg and Tr1	Phase III clinical trial, NCT04596540
CNP-103	Type 1 diabetes	Preproinsulin, GAD65, IGRP, ZnT8	Preproinsulin; glutamate decarboxylase 65 (GAD65); islet-specific glucose-6-phosphatase catalytic subunit-related protein (IGRP); zinc transporter-8 (ZnT8)	Carboxylated PLGA nanoparticles encapsulating antigens	Particle size and negative charge are optimized for selective phagocytosis by MARCO^+^ macrophages and splenic and hepatic DCs via class A scavenger receptors [129]. Uptake through these receptors serves as a clearance signal and induces a switch of APCs to a tolerogenic phenotype (CD80^low^ CD86^low^ MHCII^+^ TGF-β^+^ IL-10^+^). Ultimately this induces differentiation of CD4^+^ T cells into Foxp3^+^ Treg and Tr1 [130,131]	Phase 1b/2a clinical trial, NCT06783309
CNP-104	Primary biliary cirrhosis	PDC-E2	E2 subunit of pyruvate dehydrogenase (PDC-E2)	Carboxylated PLGA nanoparticles encapsulating antigens	Same mechanism as CNP-103	Phase 2a clinical trial, NCT05104853
TOLERVIT-MS [132]	Multiple sclerosis	MBP_13-32_, MBP_83-99_, MBP_111-129_, MBP_146-170_, PLP_139-154_, MOG_1-20_, MOG_35-55_	Myelin basic protein (MBP); myelin oligodendrocyte glycoprotein (MOG)	Vitamin D_3_-treated tolDC presenting these antigens	Induction of anergy and differentiation of CD4^+^ T cells into Foxp3^+^ Treg and Tr1	Phase I clinical trial, NCT02903537
Rheumavax [133]	Rheumatoid arthritis	Citrullinated aggrecan; citrullinated vimentin; citrullinated fibrinogen (α/β chains)	Citrullinated aggrecan; citrullinated vimentin; citrullinated fibrinogen (α/β chains)	tolDC treated with Bay11-7082 (NF-κB inhibitor) presenting these antigens	Induction of anergy and differentiation of CD4^+^ T cells into Foxp3^+^ Treg and Tr1	Phase I/II clinical trial
SGAD65_190-315_/IL-10 [134]	Type 1 diabetes	GAD65_190-315_	Glutamate decarboxylase 65 (GAD65)	DNA construct co-expressing antigen and IL-10	Induction of differentiation of CD4^+^ T cells into Foxp3^+^ Treg	Preclinical studies, mouse model
PSAB liposomes with encapsulated insulin + liraglutide [135]	Type 1 diabetes	Insulin_90-110_ (α-chain) Insulin_25-54_ (β-chain)	Insulin	Phosphatidylserine-containing liposomes mimic an apoptotic cell, and their uptake by DCs exerts a tolerizing effect. Liraglutide binds the GLP-1 receptor on pancreatic β cells, enhancing their neogenesis and proliferation	Induction of differentiation of CD4^+^ T cells into Foxp3^+^ Treg plus an increase in β-cell mass	Preclinical studies, mouse model

## 4. pMHC-Mediated Depletion of Autoreactive T-Cell Populations

### 4.1. pMHC Conjugates with Cytotoxic Agents

Beyond the induction of peripheral tolerance via anergy or Treg differentiation, recent therapeutic advancements have focused on the selective depletion of pathogenic clones. This strategy utilizes antigen-specific cytotoxic conjugates—molecular constructs where an autoantigenic peptide, presented within an MHC scaffold, is coupled to a potent effector toxin. By bypassing the need for functional reprogramming of the T-cell phenotype, these agents facilitate the direct elimination of autoreactive TCR-specific populations [136,137] (Table 4).

### 4.2. pMHC Conjugates with Oxidoreductase Motif

**Figure 5:**
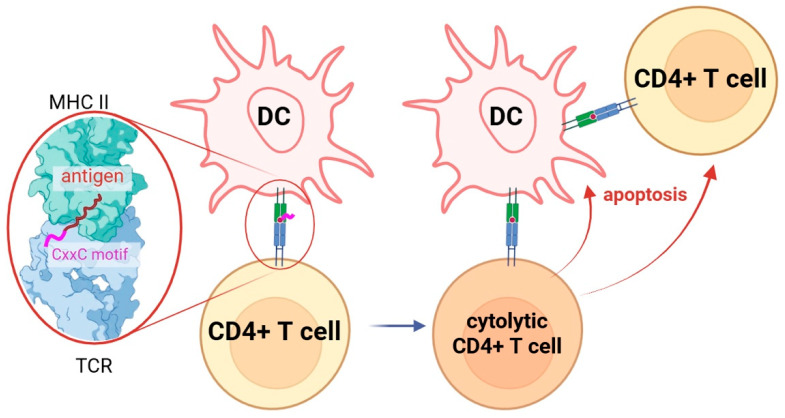
Redox-mediated differentiation of cytolytic CD4^+^ T cells. Antigens containing the CxxC thioreductase motif catalyze the reduction in surface disulfide bonds, promoting clustering of the TCR–CD4–integrin complex. This biochemical rearrangement increases the kinetic stability and robustness of the pMHC–TCR interaction, ensuring the sustained signal intensity necessary for the transition of naive T cells to a cytolytic phenotype.

A distinct example is the Imotope platform, which uses antigens engineered with an oxidoreductase CxxC motif capable of oxidizing thiol groups of amino acids to form S–S bonds [141]. The appearance of new disulfide bonds at the T-cell surface shifts CD4, TCR, and integrins into a more clustered, high-avidity state and increases the stability of pMHC–TCR complexes [136]. This results in stronger TCR-mediated signaling and conversion of the T cell to a cytolytic phenotype. Such a cytolytic T cell can induce apoptosis (via Fas–FasL and perforin–granzyme mechanisms) of immune cells involved in the autoimmune response; specifically, this localized cytotoxicity is directed toward both the antigen-presenting cells (APCs) displaying the cognate autoantigen and the pathogenic CD4^+^ effector T cells physically coupled to the APC via the pMHC–TCR interface (Figure 5, Table 4). Such a dual-targeting approach ensures the disruption of the pro-inflammatory microenvironment at the site of autoantigen recognition.

In summary, pMHC conjugates functionalized with cytotoxic or oxidoreductase domains enable the targeted depletion of pathogenic T-cell and APC populations. By preserving bystander immune function, these agents offer a more precise alternative to broad-spectrum immunosuppressants. This approach may achieve sustained remission through finite treatment courses and facilitate the development of HLA-specific therapies for chronic autoimmune diseases [137].

Compared to free antigens and native pMHC complexes, which are often limited by low efficacy and the risk of unintended immune activation, pMHC–effector conjugates ensure the targeted destruction of both pathogenic T cells and cognate APCs. Nevertheless, the safety profile of pMHC conjugates is more sensitive to epitope mapping precision; whereas tolDCs and free antigens and pMHC complexes operate within natural regulatory boundaries, the use of cytotoxic payloads creates a narrow therapeutic window, necessitating stringent HLA-restriction protocols to avoid collateral damage to healthy tissues, which was reported for CAR- and TCR-based therapies [142]. Consequently, even marginal fluctuations in dosage or systemic exposure can trigger significant off-target cytotoxicity and collateral damage to healthy tissues [137].

## 5. Therapeutic Strategies for ADs Targeting the TCR

As the primary structural determinant of the trimolecular complex, TCR functions as a molecular switch that triggers the initiation and persistence of autoimmune signaling. This section delineates therapeutic strategies designed to disrupt autoreactive signaling by directly targeting the TCR interface.

### 5.1. CAR-T Immunotherapy

At present, CAR-T therapy is becoming a highly relevant direction in personalized treatment. It already plays a major role in cancer therapy and is also applicable to autoimmunity. A CAR (chimeric antigen receptor) is an artificially engineered T-cell receptor designed to recognize specific markers or antigens. It consists of an external antigen-binding scFv domain, a hinge region that provides scFv flexibility, a transmembrane domain, anchoring the CAR in the cell membrane, and intracellular signaling domains [143]. In clinical oncology, patient-derived CD8^+^ T cells are genetically modified via lentiviral transduction to express a chimeric antigen receptor (CAR) [144]. The high-affinity interaction between the scFv domain and its cognate surface antigen (e.g., CD19, BCMA, or CD22) triggers a potent cytotoxic cascade characteristic of effector CD8^+^ lymphocytes [145,146].

In the context of autoimmunity, several CAR-T-based therapeutic platforms are under active investigation. Currently, the most clinically advanced strategy involves anti-CD19 CAR-T cells designed for the systemic depletion of the B-cell lineage. These engineered CAR-T have demonstrated significant clinical efficacy in patients with systemic lupus erythematosus (NCT06333483) and systemic sclerosis (NCT05085444). A pivotal advantage of CAR-T cells over conventional monoclonal antibodies lies in their superior chemotactic trafficking into secondary lymphoid organs, facilitating the eradication of sequestered pathogenic B-cell populations responsible for autoantibody production.

CAR-T cells targeting other surface markers on B and T cells have also been described, including CD20, CD38, BCMA, CD7, CD70, and IL23R [147,148,149,150,151]. A primary limitation of these systemic depletion strategies is the non-selective elimination of broad lymphocyte subsets, which predisposes patients to opportunistic infections and secondary immune-mediated pathologies. Consequently, the field is pivoting toward the development of precision-engineered T-cell-based platforms with modified synthetic receptors to achieve antigen-specific targeting, thereby preserving global immune surveillance while selectively neutralizing pathogenic clones.

Further therapeutic advancements focus on CAR-T cells directed specifically against the TCR complex. This strategy exploits the mutually exclusive expression of TRBC1 and TRBC2 constant domains, allowing for clonal tumor eradication while preserving a substantial fraction of the healthy T-cell repertoire [152]. Beyond its clinical success in peripheral T-cell lymphoma [153] and γδ TCR-positive malignancies [154], this approach offers a transformative blueprint for autoimmunity. Clinical remission in ankylosing spondylitis via TRBV9+ T-cell depletion [23], alongside foundational work targeting MBP-reactive clones in multiple sclerosis [155], validates the feasibility of clonotype-specific ablation. Given the documented TCR repertoire skews in RA [156], SLE [157], and type 1 diabetes [158], targeting pathogenic clonotypes represents a precise, viable alternative to global immunosuppression.

Another highly specific iteration of CAR-T immunotherapy for autoimmune diseases involves the development of Chimeric Autoantibody Receptor (CAAR) T cells and B-cell Antigen Receptor (BAR) targeted T cells. These platforms extend the principle of precision targeting by shifting the focus toward the humoral arm of the immune response. Unlike pan-B-cell depletion strategies, CAAR and BAR-T cells employ a molecular ‘baiting’ mechanism: the T cells are engineered to express specific autoantigenic epitopes that selectively engage autoreactive B cells via their cognate BCRs. This approach facilitates the elective cytolysis of pathogenic B-cell clones while maintaining the integrity of the broader, non-autoreactive B-cell repertoire, further minimizing the risks associated with systemic immunosuppression [159].

Compared to pMHC multimers and tolerogenic DCs, CAR-T therapies offer the advantage of active, irreversible elimination of pathogenic clones rather than relying on the passive induction of anergy or tolerance. As ‘living drugs,’ they provide long-term immunosurveillance and sustained remission from a single dose. However, these benefits are countered by significant safety risks, including cytokine release syndrome (CRS) and off-target toxicities, which are largely absent in multimer or tolDC strategies. Furthermore, the high manufacturing complexity and costs of CAR-T cells remain major hurdles compared to the more standardized production of pMHC-based biologics.

### 5.2. CAR-Treg Immunotherapy

By engineering CARs with “TCR-like” scFvs that specifically recognize intracellular autoantigenic peptides presented by MHC molecules, these synthetic receptors effectively substitute for the endogenous TCR. When expressed in regulatory T cells (CAR-Tregs), this approach combines the modular, potent signaling of a CAR with the exquisite specificity of a TCR, creating a programmable platform to bypass natural TCR limitations and enforce antigen-specific tolerance at the site of inflammation. Upon activation through the chimeric receptor, these CAR-Tregs secrete IL-10 and TGF-β and upregulate CTLA-4 and CD25, thereby suppressing activation and survival of effector T cells [160]. These concepts and current examples are summarized in Table 5.

### 5.3. TCR-Treg Immunotherapy

In addition, another high-precision technology is TCR-Treg (TCR-engineered Treg). This strategy utilizes the expression of pathogenic autoreactive T-cell receptors on FoxP3^+^ regulatory T cells. This approach has demonstrated therapeutic potential in models of systemic lupus erythematosus [164], multiple sclerosis [165], and type 1 diabetes [166], among other autoimmune pathologies. By redirecting the cellular response from a pro-inflammatory to a regulatory profile, these techniques facilitate the restoration of peripheral tolerance through antigen-specific immune modulation. 

In contrast to cytolytic CAR-T cells, CAR-Treg and TCR-Treg strategies focus on restoring immune homeostasis through bystander suppression rather than clonal elimination. This allows for broader control of inflammation without depleting the T-cell repertoire. However, Treg-based therapies face steeper clinical hurdles, particularly the risk of lineage instability (conversion into pro-inflammatory Th17-like cells [167]) and the technical difficulty of isolating and expanding sufficient quantities of high-purity cells. While CAR-T cells are easier to manufacture and provide rapid results, the potential for permanent immune suppression makes Tregs a more attractive, albeit more complex and logistically challenging, alternative for chronic autoimmunity.

## 6. Conclusions

Targeting the pMHC–TCR interface represents the pinnacle of precision immunology, shifting the therapeutic paradigm from broad immunosuppression to antigen-specific modulation. This review has delineated the molecular strategies—from altered peptide ligands to synthetic TCR mimetics like CAR-Tregs—that aim to decouple pathogenic signaling and restore self-tolerance. The successful preclinical and clinical validation of these modalities underscores their potential to induce durable remission by expanding regulatory phenotype cell populations while silencing autoreactive effectors.

Despite this progress, critical challenges remain, including the management of antigen epitope spreading, the high degree of HLA polymorphism, and the requirement for targeted delivery to secondary lymphoid organs. Overcoming these hurdles through the refinement of “inverse” precision therapies will be essential for the next generation of immunotherapeutics. Ultimately, the ability to reprogram the immunological synapse moves the field closer to a definitive cure for autoimmunity, rather than mere symptomatic management.

## Figures and Tables

**Table 1:** Immunotherapeutic Agents Based on soluble antigens.

Drug Name	Disease	Antigen	Origin of the Antigen	Composition	Mechanism of Action	Development Stage
Nasal insulin [81]	Type 1 diabetes (prevention)	Insulin	Insulin	Intranasal human insulin	Induction of T-cell anergy and differentiation of CD4^+^ T cells into regulatory phenotypes	Phase III clinical trial, NCT00223613
ATX-MS-1467 [82,83]	Multiple sclerosis	MBP_30-44_, MBP_83-99_, MBP_131-145_, MBP_140-154_,	Myelin basic protein (MBP)	Mixture of four MBP-derived peptides	Induction of T-cell anergy and differentiation of CD4^+^ T cells into regulatory phenotypes	Phase II clinical trial, NCT01973491
MBP8298 [84]	Multiple sclerosis	MBP_82-98_	Myelin basic protein (MBP)	Synthetic linear peptide corresponding to MBP_82–98_	Induction of T-cell anergy and differentiation of CD4^+^ T cells into regulatory phenotypes	Phase III clinical trial NCT00468611
cyclo(87–99) (Ala91,Ala96) MBP87–99 [88]	EAE (MS model)	MBP_87-99_	Myelin basic protein (MBP)	Cyclic altered peptide ligand of MBP_87–99_	Induction differentiation of CD4^+^ T cells into regulatory phenotypes	Preclinical studies, mouse model
Cyclic MOG35–55 [89]	EAE (MS model)	MOG_35–55_	Myelin oligodendrocyte glycoprotein (MOG)	Cyclic peptide analog of MOG_35–55_	Induction of T-cell anergy and differentiation of CD4^+^ T cells into regulatory phenotypes	Preclinical studies, mouse model
Glatiramer acetate (Copaxone) [90]	Multiple sclerosis	L-glutamic acid, L-lysine, L-alanine, L-tyrosine	MBP-mimetic antigenic platform	Copolymer of glutamic acid, lysine, alanine, and tyrosine	Induction of differentiation of CD4^+^ T cells into regulatory phenotypes	Approved NCT00097188
NBI-6024 [91]	Type 1 diabetes	Insulin B-chain _9–23_	Insulin	Altered peptide ligand based on insulin B (9–23)	Induction of T-cell anergy	Phase I clinical trial, NCT00873561
Jusvinza/CIGB-814 [92]	Rheumatoid arthritis	HSP60-derived epitope	HSP60	Altered peptide ligand derived from HSP60	Induction of differentiation of CD4^+^ T cells into Foxp3^+^ Treg	Phase III clinical trial, RPCEC00000404

**Table 2:** Immunotherapeutic Agents Based on pMHC complexes and nanoparticle platforms.

Drug Name	Disease	Antigen	Origin of the Antigen	Composition	Mechanism of Action	Development Stage
PVT-201 [108]	Primary biliary cirrhosis, autoimmune hepatitis	PDC-E2_166-181_, PDC-E2_82-96_, CYPD_398-412_, mFTCD_58-72_	E2 subunit of pyruvate dehydrogenase (PDC-E2), Cytochrome P450 (CYPD), formiminotransferase-cyclodeaminase/methenyltetrahydrofolate cyclohydrolase (mFTCD)	Iron–dextran pMHC nanoparticles	Induction of differentiation of autoreactive CD4^+^ T cells into Tr1 cells through multivalent engagement with pMHC.	Phase I clinical trial NCT06798454
BDC2.5mim-, CYPD_398–412_, pMOG_36–50_, PDC-E2_166–181_-IA^g7^ [109]	Type 1 diabetes	BDC2.5mim-, CYPD_398–412_, pMOG_36–50_, PDC-E2_166–181_-IA^g7^	Mimotope peptide mimicking the structure of a chromogranin A fragment (BDC2.5mim), Cytochrome P450 (CYPD), Myelin oligodendrocyte glycoprotein (MOG), E2 subunit of pyruvate dehydrogenase (PDC-E2)	Fe_3_O_4_-based nanoparticles coated with antigen–MHC II complexes	Induction of differentiation of autoreactive CD4^+^ T cells into Tr1 cells through multivalent engagement with pMHC	Preclinical studies, mouse model
BDC2.5mim–I-A^g7^ tetramers + IL-2/anti-IL-2 [110]	Type 1 diabetes	BDC2.5mim-IA^g7^	Mimotope peptide (BDC2.5mim) that mimics the structure of a chromogranin A fragment	Tetramer complexes (chromogranin A mimotope–MHC II) combined with IL-2–anti-IL-2	After formation of the IL-2–JES6-1A12 (anti-IL-2) complex, IL-2 can no longer bind the CD122^+^γc IL-2 receptor on effector T cells, whereas the CD25^+^CD122^+^γc receptor on Foxp3^+^ Treg retains the ability to bind IL-2. Thus, IL-2 in complex with the antibody selectively stimulates Foxp3^+^ Treg, while the tetramers provide antigen specificity and activation of Treg.	Preclinical studies, mouse model

**Table 4:** pMHC-Based therapeutic Agents for Autoreactive T-Cell Elimination.

Drug Name	Disease	Antigen	Origin of the Antigen	Composition	Mechanism of Action	Development Stage
MHC-CII_259_–MMAF [137]	Rheumatoid arthritis	CII_259–273_	collagen Type II (CII)	Conjugate of MHC–collagen (residues 259–273) with the cytotoxic agent MMAF, an inhibitor of tubulin polymerization	Selective elimination of autoreactive CD4^+^ T cells	Preclinical studies, cell model
H2K^d^–IGRP_201_–_214_/H2K^d^–InsB 15–23/H2D^b^–DMK 138–146 + saporin [138]	Type 1 diabetes	IGRP_201-214_, InsB _15-23_, DMK _138-146_	Islet-specific glucose 6-phosphatase catalytic subunit-related protein (IGRP), Insulin B (InsB) Dystophia myotonica kinase (DMK)	pMHC tetramer conjugates with the cytotoxic agent saporin	Selective elimination of autoreactive CD8^+^ T cells	Preclinical studies, cell model
IMCY-0141 (Imotope) [139]	Multiple sclerosis	MOG	Myelin oligodendrocyte glycoprotein (MOG)	MOG-derived antigen carrying a CxxC motif	Selective elimination of autoreactive APCs and CD4^+^ T cells	Phase II clinical trial, NCT05417269
IMCY-0098 (Imotope) [140]	Type 1 diabetes	20–34 amino acid residues of the C-peptide domain of proinsulin	Proinsulin	C20–A1 fragment of proinsulin carrying a CxxC motif	Selective elimination of autoreactive APCs and CD4^+^ T cells	Phase II clinical trial, NCT04524949

**Table 5:** CAR-Treg Therapies for Antigen-Specific Immunosuppression.

Drug Name	Disease	Antigen	Composition	Mechanism of Action	Development Stage
InsA6-CAR-Treg [161]	Type 1 diabetes	Insulin	CAR-Treg. CAR structure: Insulin-specific scFv CD8 hinge + CD8 transmembrane domain CD3ζ signaling domain	Proliferation of CAR-Treg upon activation by insulin in pancreatic islets of Langerhans and suppression of effector T cells.	Preclinical studies, mouse model
InsB10-23/IA^g7^-CAR-Treg [162]	Type 1 diabetes	InsB_10-23_/IA^g7^	CAR-Treg. CAR structure: InsB10-23/IA^g7^-specific scFv CD8 hinge + CD28 transmembrane domain CD28 and CD3ζ signaling domains	Suppression of diabetogenic CD4^+^ effector T cells and reduction in their production of pro-inflammatory cytokines.	Preclinical studies, mouse model
MOG-CAR-Treg [163]	Multiple sclerosis	MOG	CAR-Treg. CAR structure: Anti-MOG scFv CD8 hinge + CD8 transmembrane domain CD28/CD3ζ signaling domains	CAR-Treg persist within CNS lesions, suppress Th17 cells and autoantibodies, and induce long-term tolerance.	Preclinical studies, mouse mode

## Data Availability

No new data were created or analyzed in this study. Data sharing is not applicable to this article.

## Abbreviations

The following abbreviations are used in this manuscript:

ADs	Autoimmune diseases
APC	Antigen-presenting cell
APS	Antiphospholipid syndrome
BAR	B-cell Antigen Receptor
BCR	B-cell receptor
BCMA	B-cell maturation antigen
CAAR	Chimeric autoantibody receptor
CAR	Chimeric antigen receptor
CAR-T	Chimeric antigen receptor T cell
CAR-Treg	Chimeric antigen receptor regulatory T cell
CD	Cluster of differentiation
CNS	Central nervous system
CTL	Cytotoxic T lymphocyte
CTLA-4	Cytotoxic T-lymphocyte-associated protein 4
DAMPs	Damage-associated molecular patterns
DC	Dendritic cell
imDC	Immature dendritic cell
smDC	Semi-mature dendritic cell
tolDC	Tolerogenic dendritic cell
EAE	Experimental autoimmune encephalomyelitis
FoxP3	Forkhead box P3
GAD65	Glutamate decarboxylase 65
GLP-1	Glucagon-like peptide 1
GR	Glucocorticoid receptor
HLA	Human leukocyte antigen
HSPs	Heat-shock proteins
IFN-γ	Interferon gamma
IL	Interleukin
IL-2R	Interleukin-2 receptor
IGRP	Islet-specific glucose-6-phosphatase catalytic subunit-related protein
IκBα	Inhibitor of nuclear factor kappa B alpha
MBP	Myelin basic protein
MHC	Major histocompatibility complex
MHC I	Major histocompatibility complex class I
MHC II	Major histocompatibility complex class II
MMAF	Monomethyl auristatin F
MOG	Myelin oligodendrocyte glycoprotein
MS	Multiple sclerosis
NETosis	Neutrophil extracellular trap-mediated cell death
NFAT	Nuclear factor of activated T cells
NF-κB	Nuclear factor kappa B
NP	Nanoparticle
PAD	Peptidyl arginine deiminase
PDC-E2	Pyruvate dehydrogenase complex E2 subunit
PLGA	Poly(lactic-co-glycolic acid)
PLP	Proteolipid protein
PTMs	Post-translational modifications
RA	Rheumatoid arthritis
RTLs	Recombinant T-cell receptor ligands
SLE	Systemic lupus erythematosus
STAT	Signal transducer and activator of transcription
T1D	Type 1 diabetes
TCR	T-cell receptor
Tfh	Follicular helper T cell
Th1	Type 1 T helper cell
Th2	Type 2 T helper cell
Th9	Type 9 T helper cell
Th17	Type 17 T helper cell
Th22	Type 22 T helper cell
TRBC	TCR β chain constant domain
Treg	Regulatory T cell
Tr1	Type 1 regulatory T cell
TNF-α	Tumor necrosis factor alpha
ZnT8	Zinc transporter 8
